# Supplementary material for: Antipsychotic Abuse, Dependence, and Withdrawal in the Pediatric Population: A Real-World Disproportionality Analysis
Source: Biomedicines. 2022 Nov 18;10(11):2972. doi: 10.3390/biomedicines10112972 (PMC9687123; doi:10.3390/biomedicines10112972)
Supplement: Supplementary file 1 [file biomedicines-10-02972-s001.zip › biomedicines-1993283-supplementary.pdf]

Table S1. Reports of any adverse drug reaction involving risperidone, aripiprazole, quetiapine and olanzapine in children and adolescents (< 18 years old)

Age range	Risperidone	Aripiprazole	Quetiapine	Olanzapine
0-23 months	303	445	462	314
2-11 years	4,813	2,362	690	568
12-17 years	6,700	3,904	2,568	2,401
< 18 years	11,816	6,711	3,720	3,282

Table S2. Reported suspected antipsychotics in patients aged between 0 days and 23 months with abuse, dependence or withdrawal

Drugs	Number (%)
Quetiapine	75 (37.8)
Aripiprazole	30 (15.2)
Risperidone	28 (14.1)
Olanzapine	20 (10.1)
Chlorpromazine	17 (8.6)
Cyamemazine	16 (8.1)
Levomepromazine	9 (4.5)
Clozapine	8 (4.0)
Haloperidol	8 (4.0)
Amisulpride	4 (2.0)
Zuclopenthixol	3 (1.5)
Flupentixol	2 (1.0)
Ziprasidone	2 (1.0)
Chlorprothixene	1 (0.5)
Levosulpiride	1 (0.5)
Loxapine	1 (0.5)
Lurasidone	1 (0.5)
Penfluridol	1 (0.5)
Perphenazine	1 (0.5)
Pipamperone	1 (0.5)
Sulpiride	1 (0.5)
Tiapride	1 (0.5)
Melperone	1 (0.5)
Trifluoperazine	1 (0.5)

Table S3. Main Preferred Terms (PTs) reported in cases of abuse, dependence, or withdrawal in patients aged between 0 days and 23 months

Preferred Term	Number (%)
Drug withdrawal syndrome neonatal	165 (83.3)
Drug withdrawal syndrome	26 (13.1)
Drug withdrawal headache	1 (0.5)
Drug dependence	5 (2.5)
Drug abuse	2 (1.0)
Neonatal complications of substance abuse	1 (0.5)
Intentional overdose	1 (0.5)
Intentional product misuse	1 (0.5)

Table S4. Reported suspected antipsychotics in patients aged between 2 and 11 years with abuse, dependence or withdrawal

Drugs	Number (%)
Risperidone	42 (45.2)
Quetiapine	23 (24.7)
Aripiprazole	13 (14.0)
Olanzapine	10 (10.8)
Paliperidone	3 (3.2)
Cyamemazine	2 (2.2)
Chlorpromazine	1 (1.1)
Chlorprothixene	1 (1.1)
Haloperidol	1 (1.1)
Pipamperone	1 (1.1)
Clozapine	1 (1.1)
Ziprasidone	1 (1.1)

Table S5. Main PTs reported in cases of abuse, dependence, or withdrawal in patients aged between 2 years and 11 years

Preferred Term	Number (%)
Intentional overdose	26 (28.0)
Intentional product misuse	20 (21.5)
Drug withdrawal syndrome	36 (38.7)
Drug withdrawal convulsions	1 (1.1)
Drug abuse	8 (8.6)
Drug dependence	2 (1.1)

Table S6. Reported suspected antipsychotics in patients aged between 12 and 17 years with abuse, dependence or withdrawal

Drugs	Number (%)
Quetiapine	270 (36.9)
Risperidone	154 (21.0)
Olanzapine	88 (12.0)
Aripiprazole	86 (11.7)
Clozapine	29 (4.0)
Promazine	29 (4.0)
Chlorprothixene	25 (3.4)
Haloperidol	21 (2.9)
Cyamemazine	19 (2.6)
Pipamperone	16 (2.2)
Ziprasidone	15 (2.0)
Amisulpride	8 (1.1)
Levomepromazine	5 (0.7)
Lurasidone	5 (0.7)
Paliperidone	5 (0.7)
Chlorpromazine	4 (0.5)
Perazine	4 (0.5)
Sulpiride	4 (0.5)
Clotiapine	3 (0.4)
Thioridazine	3 (0.4)
Zuclopenthixol	3 (0.4)
Asenapine	2 (0.3)
Flupentixol	2 (0.3)
Fluphenazine	2 (0.3)
Loxapine	2 (0.3)
Prothipendyl	2 (0.3)
Melperone	1 (0.1)
Droperidol	1 (0.1)
Levosulpiride	1 (0.1)
Periciazine	1 (0.1)
Prochlorperazine	1 (0.1)
Tiapride	1 (0.1)

Table S7. Main PTs reported in cases of abuse, dependence, or withdrawal in patients aged between 12 years and 17 years

Preferred Term	Number (%)
Intentional overdose	440 (60.1)
Intentional product misuse	105 (14.3)
Drug abuse	112 (15.3)
Drug dependence	22 (3.0)
Substance abuse	20 (2.7)
Drug abuser	5 (0.7)
Drug use disorder	4 (0.5)
Substance abuser	3 (0.4)
Substance dependence	1 (0.1)
Substance use disorder	1 (0.1)
Drug withdrawal syndrome	62 (8.5)
Drug withdrawal headache	2 (0.3)
Drug withdrawal convulsions	1 (0.1)
